# Multifocal Fibrosing Thyroiditis: an Under-recognized Mimicker of Papillary Thyroid Carcinoma

**DOI:** 10.1007/s12022-022-09726-0

**Published:** 2022-07-11

**Authors:** Agnese Orsatti, Antonio De Leo, Federico Chiarucci, Giulia Simoncini, Nadia Cremonini, Adele Fornelli, Luca Amorosa, Thais Maloberti, Dario de Biase, Giovanni Tallini

**Affiliations:** 1 Anatomic Pathology - Department of Experimental, Diagnostic and Specialty Medicine, University of Bologna, Bologna, Italy; 2 Solid Tumor Molecular Pathology Laboratory, IRCCS Azienda Ospedaliero-Universitaria Di Bologna, Bologna, Italy; 3 Galleria del Leone 2 - Bologna - Medical Office, Bologna, Italy; 4 Anatomic Pathology Unit, Ospedale Maggiore “C.A. Pizzardi”, AUSL Bologna, Bologna, Italy; 5 Ear, Nose & Throat Unit, Ospedale Maggiore “C.A. Pizzardi”, AUSL Bologna, Bologna, Italy; 6 Department of Pharmacy and Biotechnology (FaBit), University of Bologna, Bologna, Italy

**Keywords:** Multifocal fibrosing thyroiditis, Thyroiditis, Follicular epithelial dysplasia, Reactive atypia, Papillary thyroid carcinoma

## Abstract

Multifocal fibrosing thyroiditis (MFT) is an enigmatic entity, characterized by multiple fibrotic scar-like lesions with a paucicellular fibrotic center surrounded by a cellular peripheral area with reactive-appearing follicular cell atypia and variable chronic inflammation. Although poorly recognized and likely underreported in surgical pathology, the entity is considered rare with only 65 cases to date–including the current one reported to expand on the preoperative findings of this under-recognized entity. The average age of the patients is 46.8 years (range 15–71 years), 94% are female, with female to male ratio of 15:1. Individual MFT lesions typically have a superficial location. The average number of fibrotic lesions is 15.4 (range 2–51 per MFT case). Their average size is 3.1 mm (range 0.4–15.1). MFT is a disorder of diseased thyroids, typically found postoperatively in glands removed for other reasons, such as chronic lymphocytic/Hashimoto thyroiditis (32.3%), follicular nodular disease (nodular hyperplasia) (30.1%), hyperthyroidism/diffuse hyperplasia (Graves disease) (9.2%). Intriguing is the association with papillary thyroid carcinoma–present in 38.5% of MFT cases, and particularly with sub-centimetric and multifocal papillary thyroid carcinoma, with which MFT can be confused. Cases where MFT is the only thyroid pathology (7.7%) can be preoperatively mistaken for papillary thyroid carcinoma, due to worrisome ultrasound (US) and cytologic features, both of which are here documented for the first time as a component of this article. Wider recognition of MFT and of its cytologic and ultrasound features at preoperative evaluation may reduce unnecessary thyroidectomies.

## Introduction

Multifocal fibrosing thyroiditis (MFT) is an uncommon disorder of unknown etiology and pathogenesis characterized by multiple fibrotic scar-like lesions with a paucicellular fibrotic center surrounded by a cellular peripheral area with reactive-appearing follicular cell atypia and variable chronic inflammation. The greater extent of cellularity, follicular cell atypia, and chronic inflammation at the periphery results in a typical zonal distribution of pathologic alterations in individual lesions. Described for the first time by Dr. Juan Rosai in the 1990 Tumors of the thyroid gland Armed Forces Institute of Pathology (AFIP) fascicle [1], it has been subsequently analyzed in a few publications. Although poorly recognized and likely underreported in surgical pathology, the entity is considered rare with only 65 cases to date–including the current one reported to expand on the preoperative findings of this under-recognized entity [2–5]. MFT is an enigmatic disorder, possibly a precursor of some forms of papillary thyroid carcinoma. In this article, the authors provide a comprehensive review of the literature on MFT and illustrate a case with the first description of preoperative MFT ultrasound (US) and cytologic features.

## Case Report

### Clinical History

**Fig. 1:**
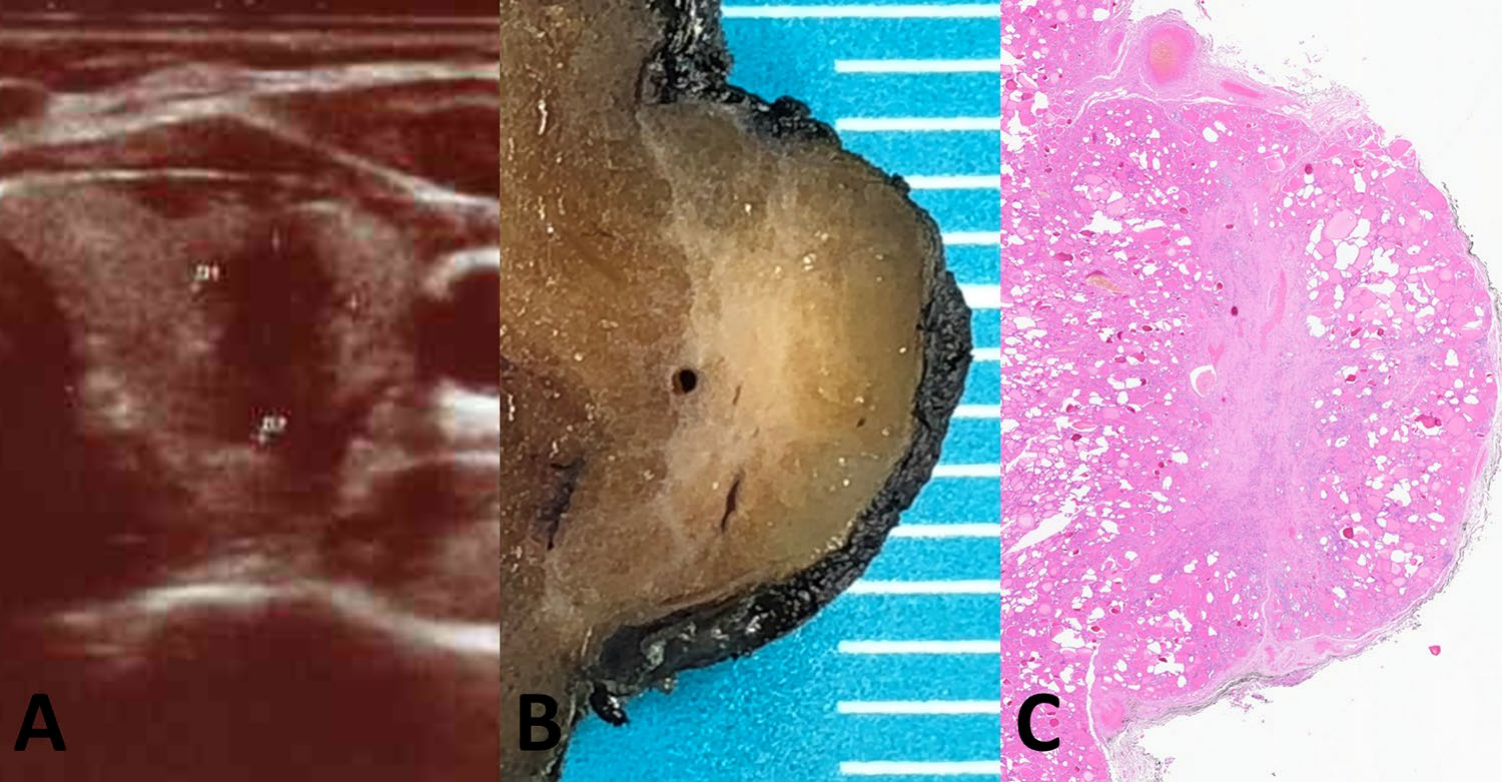
Ultrasound features and macroscopic appearance. Main MFT focus with ultrasound features worrisome for papillary carcinoma that prompted fine needle aspiration (**A**). Cut surface of the MFT focus on the surgical specimen (**B**). Low-power microscopic appearance (**C**)

A healthy 48-year-old woman underwent endocrinologic evaluation in October 2021 for family history of thyroid carcinoma since her mother had been treated for papillary carcinoma 30 years before. Endocrine function tests were within normal limits, serum antibodies negative, and the clinical setting unremarkable. Neck US–performed with an Esaote MyLabGamma scanner, probe SL 1543–detected a markedly hypoechoic, inhomogeneous nodule with irregular borders in the lower portion of the left lobe of the thyroid gland measuring 8 × 9.6 × 13.7 mm (Fig. 1). The US appearance, compatible with EU-TIRADS 5 [6] /High suspicion American Thyroid Association (ATA) category [7], was very worrisome for papillary thyroid carcinoma and a US-guided fine-needle aspiration biopsy (FNAB) was performed.

### Cytopathology

**Fig. 2:**
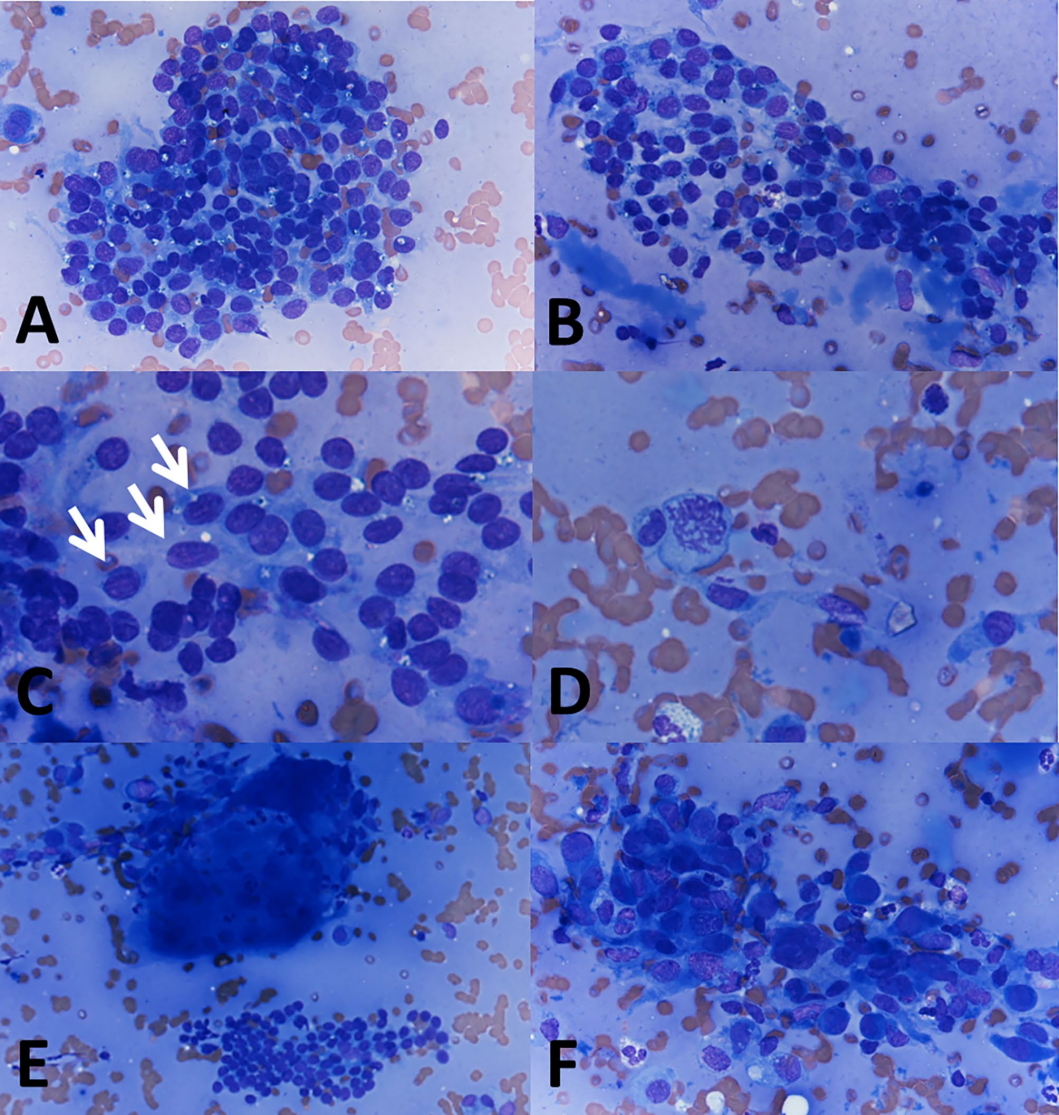
Fine needle aspiration cytology. Scant colloid and small sheets of thyroid cells with anisonucleosis (**A**, 400 ×). Pseudo-papillary cluster (**B**, 400 ×). Mildly atypical cells, some with nuclear grooves (**C**, 600 × ; arrows). Occasional mitosis (**D**, 600 ×). Multinucleated giant cell (**E**, 200 ×). Macrophages intermixed with follicular cells showing reactive atypia (**F**, 400 ×)

The FNAB smear was bloody and hypocellular, with scant colloid and small sheets of thyroid cells showing anisonucleosis, a few nuclear grooves, and rare mitoses. Occasional pseudo-papillary clusters, multinucleated giant cells, and scattered groups of atypical follicular thyroid cells intermixed with macrophages were also present (Fig. 2).

The cytopathology report was diagnosed as suspicious for papillary thyroid carcinoma, TIR4 according to the Italian reporting system for thyroid cytology [8, 9] (equivalent to Bethesda V and to Thy4-British Thyroid Association categories). The patient underwent total thyroidectomy in December 2021. The post-operative course was uneventful.

### Surgical Pathology

The resected thyroid gland weighed 20 g and showed a regular outline. On sectioning, one irregular, stellate, fibrous-appearing nodule measuring 1 cm was identified in the lower portion of the left thyroid lobe, confirming the US finding (Fig. 1B). Four additional fibrous nodules were identified, three in the left lobe (measuring 6 mm, 4 mm, and 3 mm), the fourth in the right lobe (measuring 5 mm): all were smaller, but grossly very similar to the larger nodule in the lower left lobe.

**Fig. 3:**
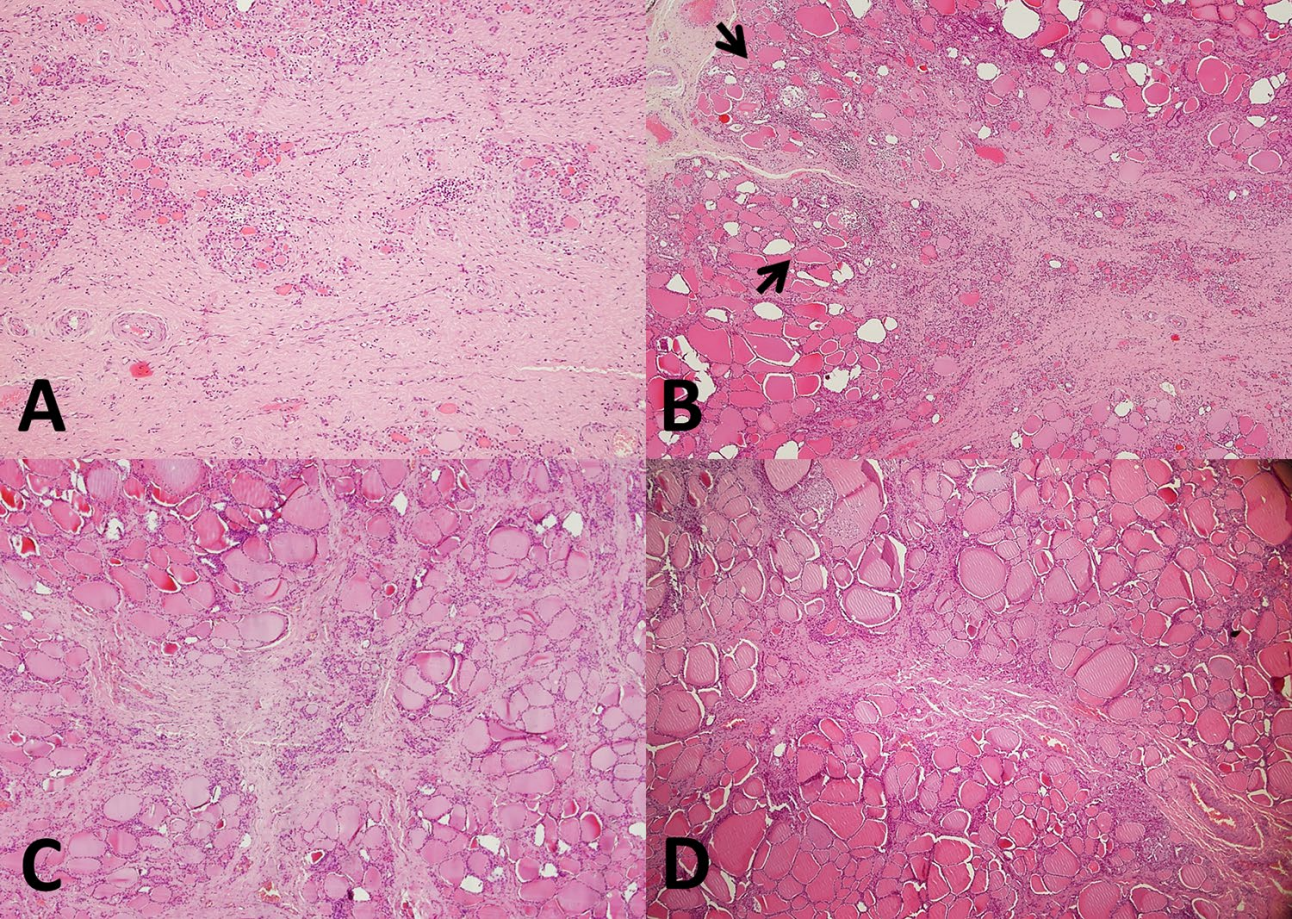
Histologic features. Zonal distribution of pathologic alterations in the largest 1 cm lesion, with paucicellular fibrotic center (**A**, 100 ×) and cellular peripheral area with foci of chronic inflammation (**B**, 40 × ; arrows). Additional fibrosing thyroiditis foci have the same histologic features of the largest focus: 4 mm (**C**, 40 ×) and 3 mm (**D**, 40 ×) lesions from the left lobe

**Fig. 4:**
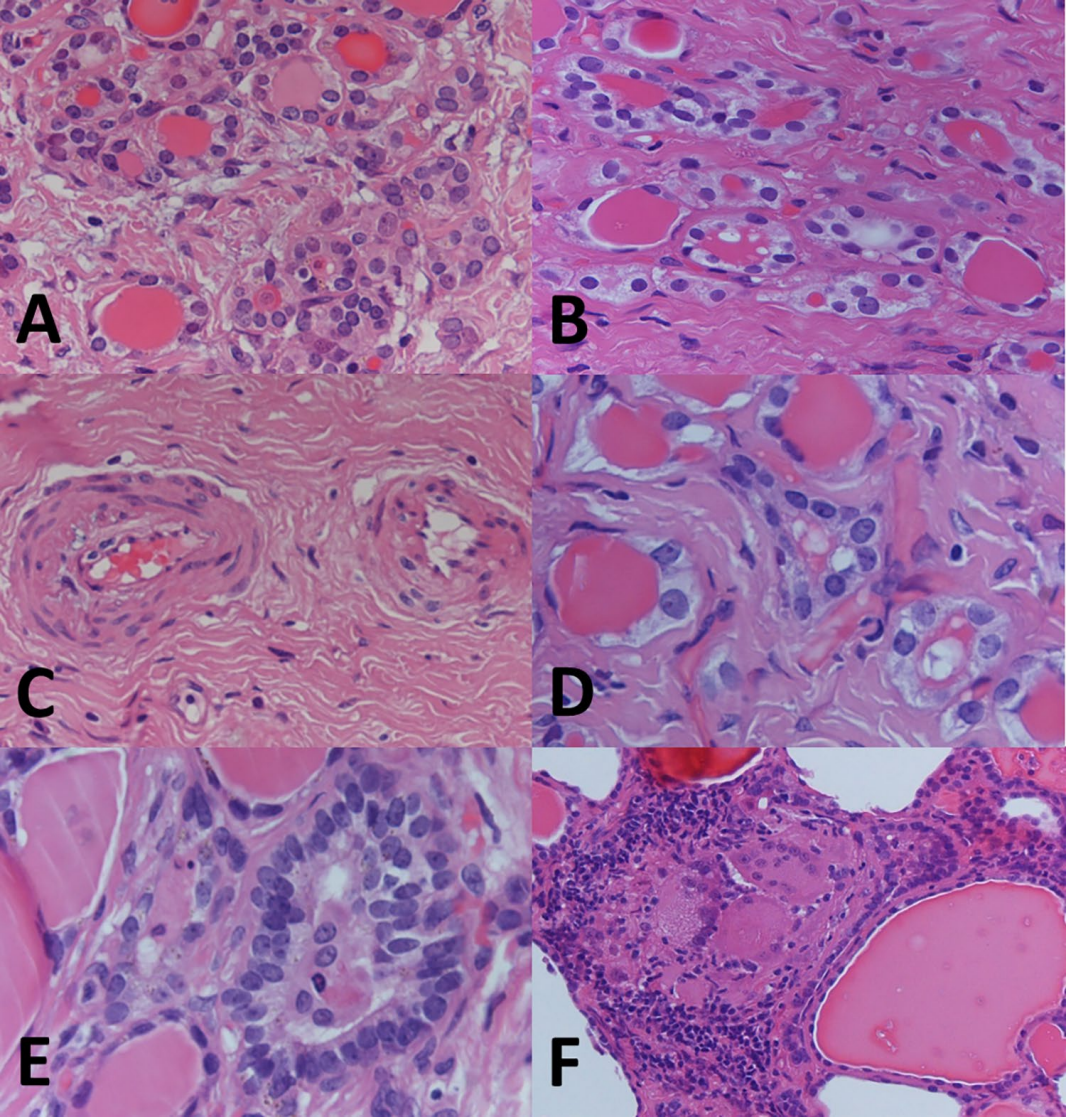
Histologic features. Small follicles with scant colloid (**A**, 400 ×), pseudoinfiltrative follicles entrapped in fibrous tissue (**B**, 400 ×), and vessels with prominent walls (**C**, 400 ×) at the center of the fibrotic area. Reactive follicular cell atypia (**D**, 600 ×), elongated follicles lined by crowded follicular cells with overlapping nuclei (**E**, 600 ×), and multinucleated giant cells with chronic inflammation (**F**, 200 ×) at the periphery of the central fibrotic core

Histologic examination of the larger nodule revealed a ramified fibrous lesion that at low power simulated papillary thyroid carcinoma with sclerosis (Fig. 1C). At higher magnification, a zonal distribution of pathologic changes was readily apparent (Fig. 3). Clusters of small follicles lined by bland, benign-appearing follicular cells and containing scant colloid were embedded in a paucicellular central fibrous area (Figs. 3A and 4A), some of the follicles entrapped in the fibrous tissue showed pseudoinfiltrative features (Fig. 4B). Scattered vessels–some ectatic, others with prominent walls–were also present in the fibrotic center (Fig. 4C). The periphery of the fibrotic core was cellular (Fig. 3B), due to foci of inflammation and an increased number of follicles. These follicles were lined by cells with mild atypia in the form of nuclear enlargement, some degree of chromatin clearing, and minor irregularities of the nuclear contour (Fig. 4D). Few mitoses were noted in some of these follicles, consistent with what observed in the FNAB (Fig. 2D). Some of the follicles were elongated, with their main axis perpendicular to the MFT central fibrotic core, and lined by cells with mild chromatin clearing and overlapping nuclei (Fig. 4E). Foci of predominantly chronic inflammation with histiocytes and scattered multinucleated giant cells were also present at the periphery of the fibrotic core (Figs. 3B and 4F). The other fibrous nodules noted grossly had virtually identical microscopic features to the 1 cm nodule (Fig. 3C, D). Those in the left lobe had a stellate configuration, that in the right lobe had a fibrous band-like appearance. Focal, predominantly chronic inflammation with scattered multinucleated giant cells was present at the periphery of all four additional MFT foci. All five foci had a superficial location, close to the outer surface of the thyroid gland (the thyroid “capsule”). The overall findings were typical of multifocal fibrosing thyroiditis (MFT). After careful evaluation and submission of the entire gland for histologic examination no papillary thyroid carcinoma was identified, nor was there any other relevant pathologic alteration.

Immunohistochemistry was negative for mutation-specific BRAF V600E (VE1) monoclonal antibody and Pan-TRK. Molecular analysis performed on formalin-fixed paraffin-embedded material dissected from the largest MFT focus using a laboratory-developed next-generation sequencing panel [10] was negative for mutations in *BRAF* (LRG_299), *HRAS* (LRG_506), *KRAS* (LRG_344), *NRAS* (LRG_92), *PIK3CA* (LRG_310), *PTEN* (LRG_311), *TERT* (LRG_343) (promoter), and *TP53* (LRG_321).

## Review of the Literature and Discussion of the Case

### Epidemiology

We know little of MFT. Since its original description in 1990 [1], the only large series based on Dr. Rosai’s consultation files was reported by Fellegara and Rosai in 2015 [4]. MFT is not common, with an incidence estimated at 0.0275 per year based on the number of cases reviewed by Dr. Rosai in a 20-year period (1989–2009) consultation practice [4]. Because of the fibrosis and the follicular cell atypia, MFT can simulate papillary carcinoma macroscopically and sometimes also microscopically. Since only problematic cases have been sent for second opinion, MFT is very likely under-reported and there is little awareness of the entity among practicing pathologists. It is possible that in the diagnostic routine some MFT cases have been misdiagnosed as papillary thyroid carcinoma. Table 1 is a summary of the features of all reported MFT cases. MFT is vastly more common in females, which accounts for 94% (60/64) of all known cases, affects patients with a mean age of 46.8 years, and a wide age range (15 to 71 years).

**Table 1:** Clinicopathologic features of reported Multifocal fibrosing thyroiditis cases

**Reference (*n*: number of cases)**	**Age, range (mean)**	**Sex (F:M)**	**Size of MFT lesions in mm, range (mean)**	**Number of MFT foci, range (mean)**	**MFT lymphoid infiltration (percentage of cases)**	**MFT multinucleated histiocytes (percentage of cases)**	**Thyroid pathology associated with MFT (Percentage of cases); average number of papillary carcinoma foci in MFT cases with PMC or PTC**^**c**^	**BRAF mutation in MFT (percentage of cases)**
Poli F et al. Int J Surg Pathol. 2009;17(2):144 (*n* = 1)	64	F	NA	NA	NA	NA	FND (NH) 1/1(100)	NA
Frank R et al. Endocr Pathol. 2014;25(3):236-40^a^ (*n* = 7)	33–51 (42.7)	F:7;M:0	NA	NA	NA	NA	CL/HT: 7/7 (100) PMC: 2/7 (28.6) and PTC: 5/7 (71.4); 1.4	0/7 (0)
Fellegara G, Rosai J. Am J Surg Pathol. 2015;39(3):416–24 (*n* = 55)^b^	15–71 (47.0)	F:51; M:4 (12.7)	0.4–15.1 (3.0)	2–51 (16.0)	38/38 (100) 14/38 (36.8) infiltration moderate-to-severe	21/38 (55.2) 5/38 (13.2) numerous multinucleated histiocytes	FND (NH): 19/55 (34.5) CL/HT: 14/55 (25.4) HT/GD: 6/55 (10.9) FA: 4/55 (7.3) FVPTC/NIFTP: 4/55 (7.3) FC: 2/55 (3.6) OC: 1/55 (1.8) PMC 16/55 (29.1) and PTC: 1/55 (1.8); 1.4 NTP: 4/55 (7.3)	NA
Fellegara G, Rosai J. Am J Surg Pathol. 2015;39(6):870 (*n* = 1)	NA	NA	NA	3	NA	NA	PMC 1/1(100)	NA
Current case (*n* = 1)	48	F	3–10 (5.6)	5 (5)	1/1 (100) moderate infiltration	1/1 (100)	NTP 1/1(100)	0/1 (0)
**Total (*n* = 65)**	**15**–**71 (46.8)**	**F:60; M:4** **(15.0)**	**0.4**–**15.1 (3.1)**	**2**–**51 (15.4)**	**39/39 (100)**	**22/39 (56.4)**	**FND(NH): 20/65 (30.1)** **CL/HT: 21/65 (32.3)** **HT/GD: 6/65 (9.2)** **FA: 4/65 (6.1)** **FVPTC/NIFTP: 4/65 (6.1)** **FC: 2/65 (3.1)** **OC: 1/65 (1.5)** **PMC 19/65 (29.2) and PTC: 6/65 (9.2); 1.5** **NTP: 5/65 (7.7)**	**0/8 (0)**

*F* female, *M* male, *MFT* multifocal fibrosing thyroiditis, *mm* millimeters, *PMC* papillary thyroid microcarcinoma, *PTC* conventional papillary thyroid carcinoma larger than one centimeter, *FND*(*NH*) follicular nodular disease (nodular hyperplasia), *CL*/*HT* chronic lymphocytic/Hashimoto thyroiditis, *HT*/*GD* hyperthyroidism/Graves disease, *FA* follicular adenoma, *FVPTC*/*NIFTP* follicular variant PTC/noninvasive follicular thyroid neoplasm with papillary-like nuclear features, *FC* follicular carcinoma (encapsulated), *OC* oncocytic carcinoma, *NTP* no thyroid pathology (neither PMC nor other pathology), *NA* not available

^a^Only MFT associated with PTC were selected for the study

^b^In 38 of the 55 cases slides were available for review

^c^More than one alteration (PMC or other pathology) may coexist in a given case

### MFT Is a Disorder of Diseased Thyroids

MFT is typically found incidentally, postoperatively, in thyroidectomy specimens performed for other reasons, with chronic lymphocytic/Hashimoto thyroiditis (32.3%) and follicular nodular disease (nodular hyperplasia) (30.1%) being the most common associated conditions. Hyperthyroidism/diffuse hyperplasia (Graves disease) is found in 9.2% of MFT cases. Very intriguing is the high proportion of cases associated with papillary carcinoma –38.5% (25/65 cases)–and in particular with papillary microcarcinoma (29.2%, 19/65 cases), as well as the fact that these papillary carcinomas are frequently multicentric, with an average number of 1.5 foci per MFT case. Follicular patterned thyroid tumors, benign or malignant, associated with MFT include follicular adenoma (6.1%), follicular variant papillary carcinoma/NIFTP (6.1%), and follicular thyroid carcinoma (3.1%). Thus, MFT is a disorder of pathologically altered thyroids. Even considering that some of the alterations reported in association with MFT may coexist with each other (e.g., papillary microcarcinoma and Hashimoto thyroiditis), in only 5 of 65 MFT cases (7.7%) no other pathology was reported in the gland (Table 1).

### Pathology and Differential Diagnosis

**Fig. 5:**
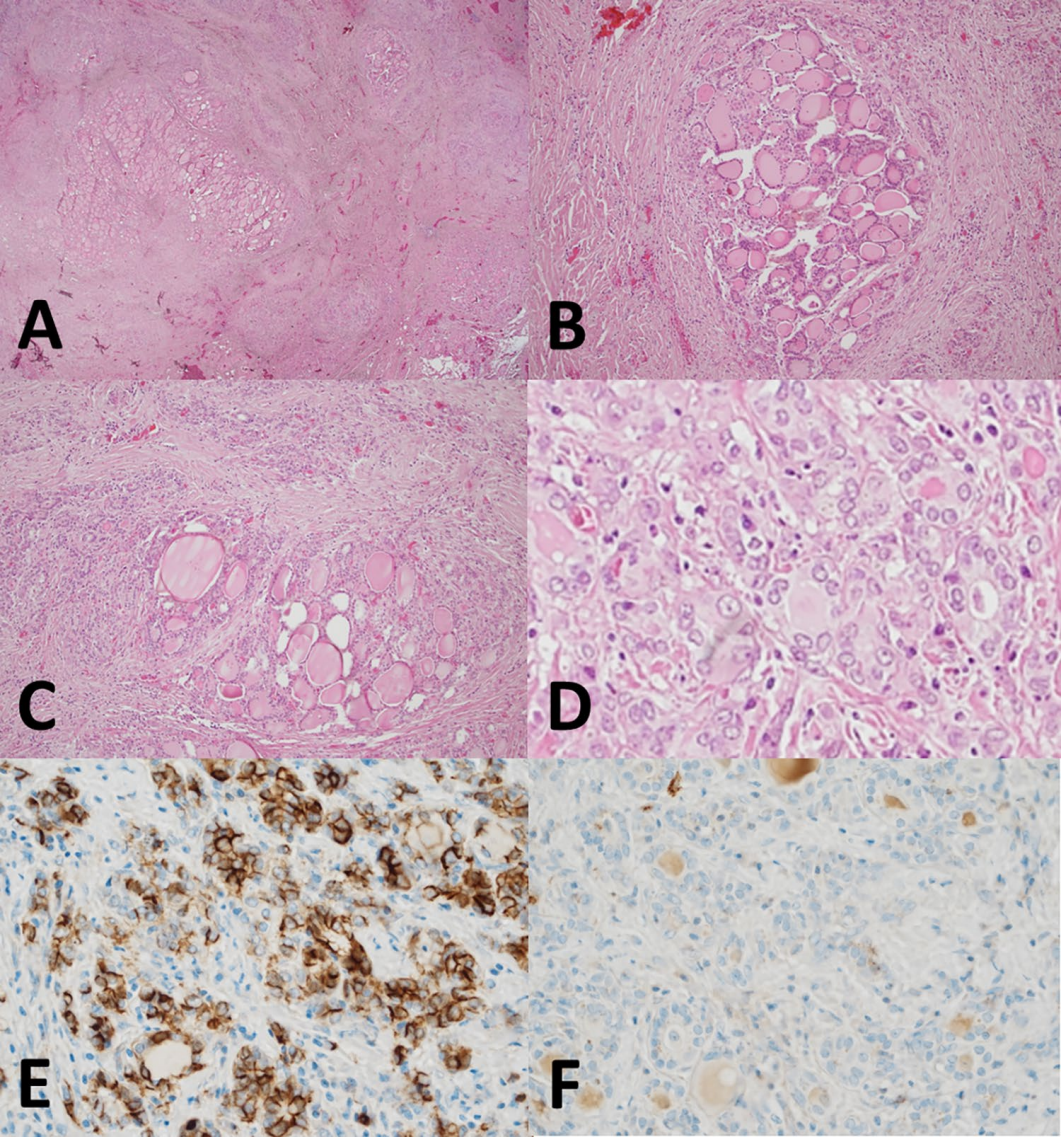
Histologic features. Advanced MFT found incidentally in a thyroid gland removed for a follicular adenoma. The patient’s clinical setting was unremarkable and serum anti-thyroid antibodies were negative. Confluence of fibrotic foci replace large portions of the thyroid parenchyma, separating it into nodules and resulting in a cirrhosis-like pattern (**A**, 20 × ; **B**, 100 × ; **C**, 100 ×). Follicles entrapped in fibrous tissue with enlarged nuclei and chromatin clearing mimicking papillary thyroid carcinoma (**D**, 600 ×). By immunohistochemistry, atypical follicular cells show partial CD-56 loss (**E**, 400 ×), HBME-1 is not overexpressed (**F**, 400 ×)

MFT typically has a superficial–as opposed to intraparenchymal/intrathyroidal–location [4]. As the “multifocal” term implies, the condition presents with a variable number of lesions within the thyroid gland, as high as 51, the average number of lesions being 15.4 per case. The size of individual fibrotic foci is also variable, some are larger than 1 cm, but most are small, with an average size of 3.1 mm. The zonal distribution of histologic alterations, with a central fibrous core and a peripheral cellular and variably inflamed area is typical of MFT (Fig. 3). However, in advanced MFT forms, where wide fibrous bands separate residual parenchyma in nodules producing in the thyroid a cirrhotic-like pattern, it may be lost (Fig. 5). In these advanced cases, follicular cells outside the better-preserved nodules and entrapped within the fibrous bands may display conspicuous cytologic atypia (Fig. 5D).

**Fig. 6:**
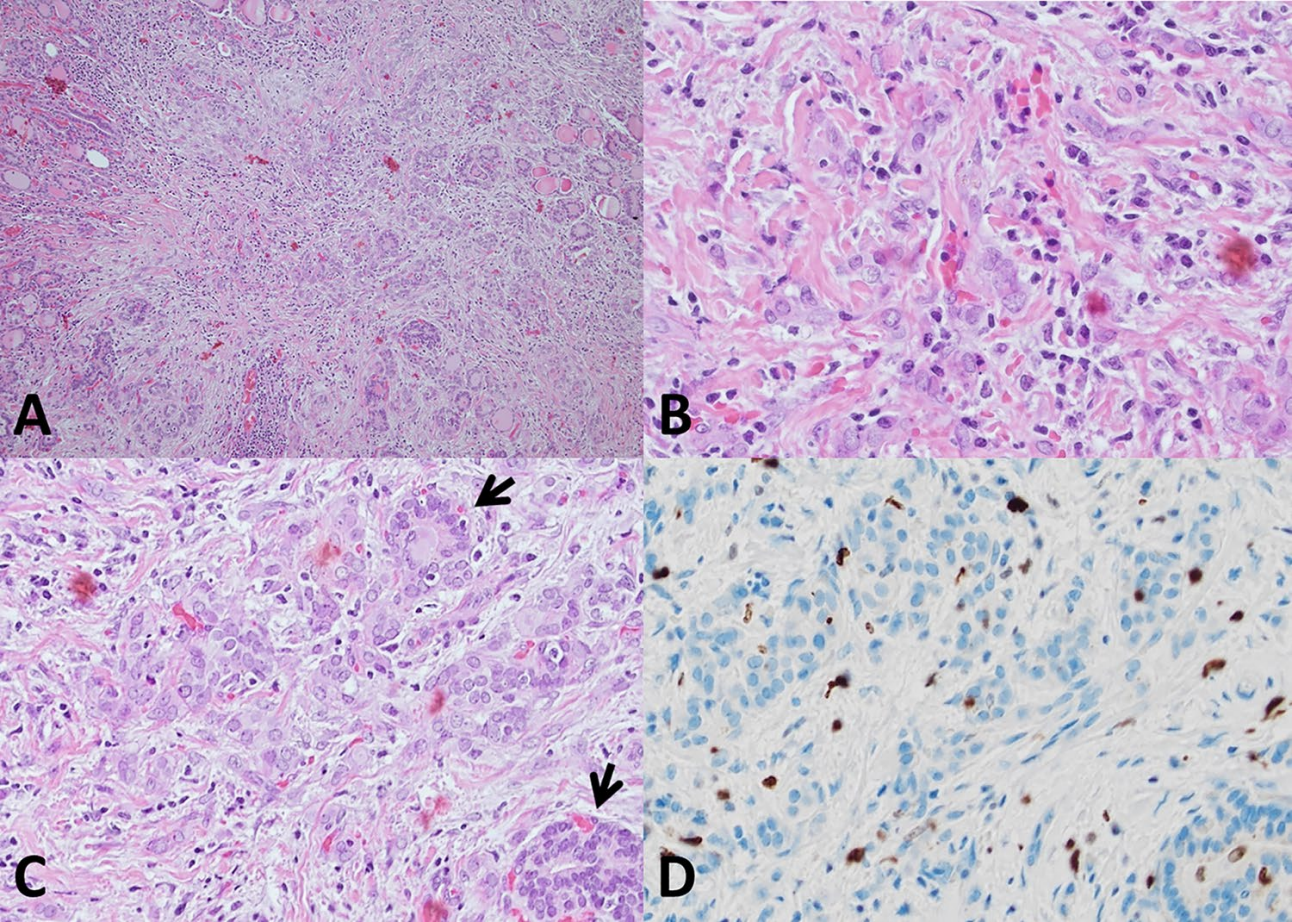
Histologic features. Millimetric MFT focus close to the outer surface of the thyroid gland mimicking at low magnification so-called occult sclerosing papillary thyroid carcinoma (**A**, 100 ×): MFT was an incidental diagnosis in a thyroid gland removed for other thyroid pathology. Follicles entrapped in the central fibrous MFT core show pseudoinfiltrative growth (**B**, 400 ×). Elongated follicles with their main axis perpendicular to the fibrotic center lined by crowded follicular cells (**C**, 200 × ; arrows). Ki67 positivity is largely limited to infiltrating lymphocytes (**D**, 400 ×)

The main differential diagnosis of MFT is with papillary thyroid carcinoma, specifically microcarcinoma, of which it represents an under-appreciated mimicker. The differential diagnosis may be particularly problematic in the case of small superficial MFT foci in which the zonal distribution of pathologic alterations is poorly evident given the limited size of the lesion (Fig. 6). Immunohistochemistry for papillary thyroid carcinoma markers such as HBME-1, cytokeratin 19, Galectin-3 (all overexpressed in papillary thyroid carcinoma), and CD56 (lost in conventional papillary thyroid carcinoma) [11] may be helpful, but results are often inconsistent since there may be partial HBME-1, cytokeratin 19, Galectin-3 expression, and partial CD56 loss (Fig. 5E, F). Ki67 is expressed in infiltrating lymphocytes, while follicular cells have little proliferative activity (Fig. 6D). Papillary thyroid carcinoma with fibromatosis/fasciitis-like stroma should be mentioned in the differential diagnosis. It is distinguished from MFT because of its clear-cut neoplastic appearance and because of nuclear beta-catenin accumulation by immunohistochemistry due to frequent CTNNB1 (beta-catenin gene) mutation in the mesenchymal component of the tumor.

The focal nature and the zonal distribution of histologic alterations in MFT generally distinguish it from both the fibrosing variant of Hashimoto thyroiditis and from Riedel thyroiditis. Furthermore, and to the point, the absence of oncocytic changes and of active fibroplasia tells apart MFT from the fibrosing variant of Hashimoto thyroiditis and from Riedel thyroiditis, respectively. Subacute (De Quervain) thyroiditis is often multifocal, but lacks a zonal distribution of histologic alterations, has numerous giant cell granulomas and foci of acute inflammation. A wedge-shaped configuration, florid inflammation, and the presence of hemosiderin deposits distinguish fine needle aspiration tract-related alterations from MFT.

Although MFT is usually found in pathologically altered thyroids removed for other reasons, the case documented here demonstrates how in the absence of co-existing thyroid pathology large MFT fibrous lesions simulating papillary thyroid carcinoma may cause an unnecessary thyroidectomy. The US features of MFT are here documented for the first time. Lesions appear on US as markedly hypoechoic and irregular with an uneven, infiltrative-looking outline, as illustrated in Fig. 1A. On FNAB cytology–also documented here for the first time–the diagnosis of suspicious for papillary carcinoma (TIR4/Bethesda V/Thy4-BTA) stems from the cytologic atypia and the nuclear groves in the follicular cells, as illustrated in Fig. 2.

The fact that MFT can be mistaken for papillary carcinoma during preoperative evaluation is not surprising. The gross appearance, and sometimes even the low power histology of a given MFT focus, simulates papillary carcinoma. The histologic distinction is usually (although not always) easy after careful assessment of the architecture of the lesion at medium to high magnification. Thus, US imaging reflects the worrisome gross appearance of the lesions. FNAB cytology does not allow to evaluate the architecture, and cytologic follicular cell atypia found in the sclerotic foci can easily be mistaken for papillary carcinoma in FNAB cytology specimens, as occurred with our patient. Indeed, evidence indicates that there is an overlap with some of the FNAB findings for papillary thyroid carcinoma (Table 2). Potential pitfalls include the presence of occasional grooves, irregular pseudopapillary clusters, and mitoses. An additional element that may cause confusion is the presence of multinucleated giant cells–found at the periphery of the fibrotic core in our case and reported in 56.4% of MFT cases (Table 1). The absence of significant chromatin clearing, nuclear molding, and of intranuclear inclusions are most useful to tell apart MFT from papillary thyroid carcinoma on FNAB cytology specimens.

**Table 2:** FNA cytology of multifocal fibrosing thyroiditis vs. classic papillary thyroid carcinoma

	**Multifocal fibrosing thyroiditis**	**Classic papillary carcinoma**
Cellularity	+	+ + +
Colloid	+	+
Papillae	− (Pseudopapillary clusters may be present)	+ + +
Microfollicles	+	+ / −
Nuclear Molding	−	+ +
Nuclear enlargement	+	+ + +
Elongated Nuclei	+	+ + +
Nuclear grooves	+	+ + +
Intranuclear inclusions	−	+ + +
Chromatin clearing	+ / −	+ + +
Mitoses	+	+ / −
Macrophages	+	+ / −
Giant cells	+	+ +

### Etiology and Pathogenesis

The etiology and pathogenesis of MFT are currently unknown, and unclear is its relationship with papillary thyroid carcinoma, with which it is commonly associated. It is likely that non-invasive follicular patterned tumors–such as follicular adenoma and encapsulated non-invasive follicular variant papillary carcinoma–are precursors to follicular carcinoma and invasive encapsulated follicular variant papillary carcinoma, respectively, based on the molecular alterations they share, and on the notion of multifocal intranodular progression of genetic changes from histologically benign (i.e., non-invasive) to histologically malignant (i.e., invasive) tumors [12–17]. However, the precursor lesions to conventional (i.e., non-follicular patterned) papillary carcinoma have remained elusive. The concept of follicular epithelial dysplasia has been proposed for the foci of papillary thyroid carcinoma-like cytologic atypia and architectural distortion associated with lymphocytic thyroiditis based on the expression pattern of papillary carcinoma immunohistochemical markers (HBME-1, cytokeratin 19, Galectin-3) [17–19], while low-level CCDC6-RET and NCOA4-RET fusion in Hashimoto thyroiditis-related follicular cells also points to a possible follicular cell atypia-dysplasia-to-carcinoma progression sequence in the setting of lymphocytic thyroiditis [20]. Furthermore, a mutagenic process affecting *BRAF* exon 15 is suggested by the occurrence of *BRAF* non p.V600E mutations of little oncogenic potential in the non-neoplastic thyroid surrounding *BRAF* p.V600E mutated papillary thyroid carcinomas, sometimes in foci of follicular cell atypia, and in the absence of lymphocytic thyroiditis [21]. In this context, the possibility that MFT–or at least some of its foci in a given case–may precede the development of papillary carcinoma is tantalizing and may be further supported by partial alteration of HBME-1, cytokeratin 19, Galectin-3, and CD56 expression patterns.

**Fig. 7:**
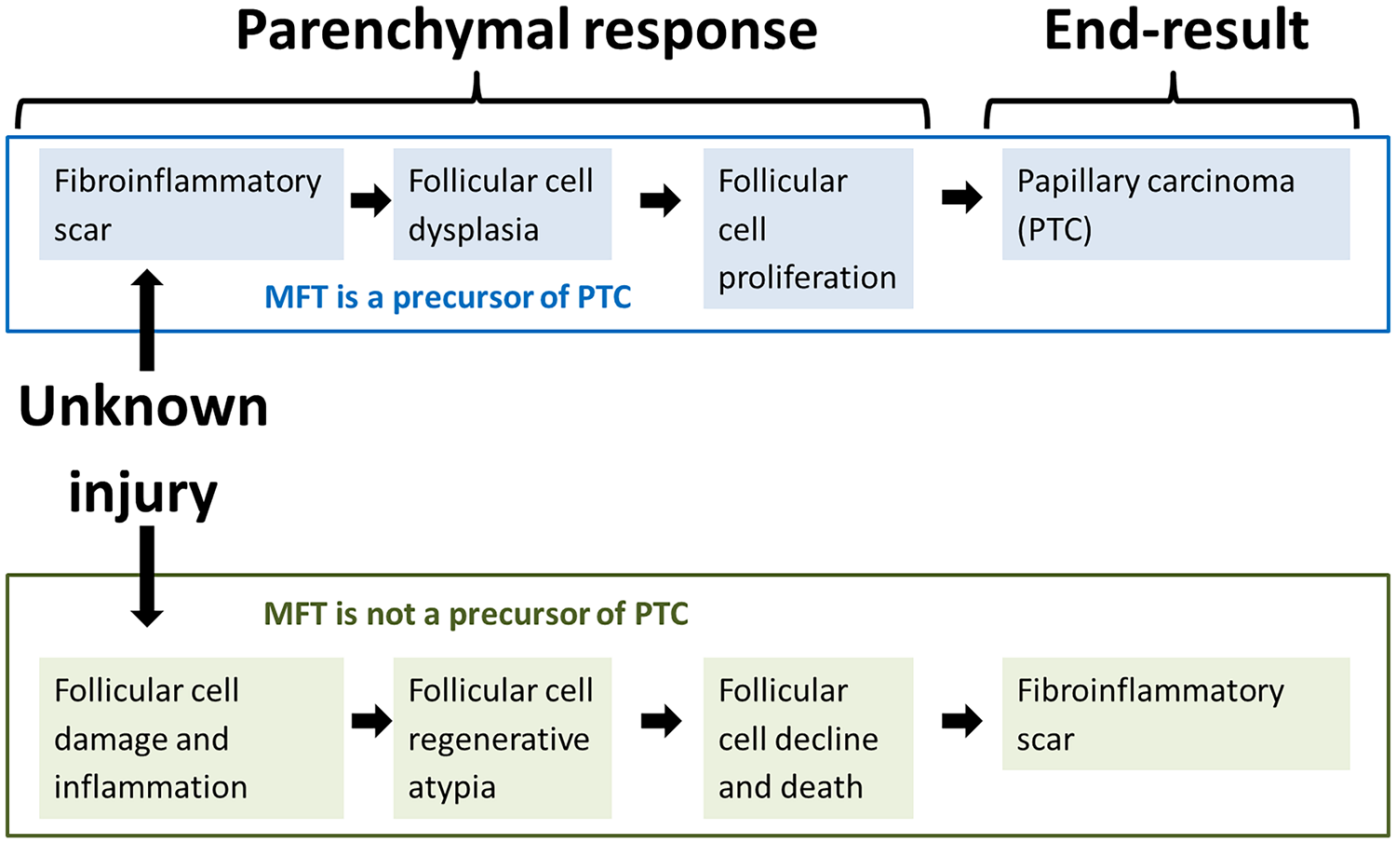
Views on the pathogenesis of Multifocal fibrosing thyroiditis based on divergent interpretation of histologic findings

Given the frequent superficial location of MFT and the high prevalence of synchronous papillary carcinoma (particularly of multifocal papillary carcinoma), Fellegara and Rosai [4] have hypothesized that MFT may be a preneoplastic condition precursor of papillary carcinoma, or at least of some of its forms, such as those originally described as occult sclerosing variant of papillary thyroid carcinoma [22–24], and that currently represent an important subgroup of subcentimeter papillary carcinomas with frequent invasive behavior [25, 26]. According to Fellegara and Rosai, additional circumstantial evidence for a link between MFT and papillary carcinoma is the similar association between fibrosis and malignancy observed in other organs, such as that between radial scar and ductal carcinoma of the breast, and between pulmonary fibrosis and lung adenocarcinoma (so-called scar-cancer) [2, 4]. Following this view, the fibrosis precedes the onset of the tumor and, by inducing cellular alterations, promotes its development, the end result being papillary carcinoma (Fig. 7). Similar hypotheses have also been discussed in the context of chronic lymphocytic thyroiditis-related follicular cell dysplasia [18].

At present, no molecular alterations have been found in seven cases of MFT all of which were associated with papillary thyroid carcinoma elsewhere in the thyroid gland [3], nor were they found in our case after immunohistochemical analysis and molecular evaluation with a next-generation sequencing panel. Although *absence of proof is not proof of absence*, an alternative (we should say less exciting) scenario is that MFT simply represents the end-result of follicular cell damage with inflammation and parenchymal scarring [3, 4]. This brings up the issue of the potential context in which the follicular cell damage occurs. At present, we have no clues as to its causes. MFT clearly represents the outcome of a yet undefined, localized, but multifocal, parenchymal injury resulting in the scar-like configuration typical of MFT. Chronic lymphocytic/Hashimoto thyroiditis–found in approximately one-third of MFT cases (Table 1)–may be the source of the injury [3, 4]. Given the multifocal nature of MFT, ischemia should also be considered as a possible cause of parenchymal injury; however, in our case and in the others previously reported, there is no evidence of vasculitis or vascular injury. Based on the common presence of giant cells found in all MFT foci of our case and more than half of those in the Fellegara and Rosai series [4], another possibility to be explored is that MFT may represent a late, burnt out/scarred stage of subacute (De Quervain) thyroiditis. However, there was no clinical evidence of such disorder in our patient, nor has the association been reported in other cases.

## Conclusion

This article provides a comprehensive overview of evidence-based data on MFT, and also reports for the first time its US and cytologic correlates. MFT is not a fascinating but clinically irrelevant thyroid curiosity. It can be misinterpreted as papillary thyroid carcinoma not only in surgically resected specimens, but also preoperatively after US examination and FNAB cytology specimens. Therefore, evidence mandates that MFT should be added to the list of lesions that can simulate papillary thyroid carcinoma during the preoperative evaluation of thyroid nodules. Wider recognition of the entity and of its preoperative US and cytologic features may improve patient care and reduce unnecessary thyroidectomies.

## Data Availability

All available data are contained within the article.
